# Mesenchymal Stem/Stromal Cell Therapeutic Features: The Bridge between the Bench and the Clinic

**DOI:** 10.3390/jcm10050905

**Published:** 2021-02-25

**Authors:** Makram Merimi, Philippe Lewalle, Nathalie Meuleman, Douâa Moussa Agha, Hoda El-Kehdy, Fatima Bouhtit, Sara Ayoub, Arsène Burny, Hassan Fahmi, Laurence Lagneaux, Mehdi Najar

**Affiliations:** 1 Laboratory of Experimental Hematology, Institut Jules Bordet, Université Libre de Bruxelles, 1000 Bruxelles, Belgium; makram.merimi.cri@gmail.com (M.M.); philippe.lewalle@bordet.be (P.L.); nathalie.meuleman@bordet.be (N.M.); douaa.moussa@gmail.com (D.M.A.); bouhtitfatima@gmail.com (F.B.); 2 Genetics and Immune Cell Therapy Unit, LBBES Laboratory, Faculty of Sciences, University Mohammed Premier, Oujda 60000, Morocco; 3 Laboratory of Pediatric Hepatology and Cell Therapy, UCLouvain, Institut de Recherche Expérimentale et Clinique, 1200 Brussels, Belgium; Hodakehdy@yahoo.fr; 4 Department of Prosthodontics, Faculty of Dental Medicine, Lebanese University, Hadath, 10999 Beirut, Lebanon; Sara.ayoub.1@ul.edu.lb; 5 Laboratory of Molecular and Cellular Biology, Faculté Universitaire des Sciences Agronomiques de Gembloux, 5030 Gembloux, Belgium; arsene.burny@guest.uliege.be; 6 Osteoarthritis Research Unit, University of Montreal Hospital Research Center, Montreal, QC H2X 0A9, Canada; h.fahmi@umontreal.ca; 7 Laboratory of Clinical Cell Therapy, Institut Jules Bordet, Université Libre de Bruxelles, 1070 Brussels, Belgium; laurence.lagneaux@bordet.bec

Mesenchymal stem/stromal cells (MSCs) are considered a relevant therapeutic product for various clinical applications. They have generated great interest as cell therapies for a range of inflammatory and autoimmune conditions [1]. Residing in different niches, these progenitor cells are involved in tissue homeostasis. Though largely debated, the presence of circulating endogenous MSCs (native MSCs) has been reported in multiple pathophysiological conditions, but the significance of such cell circulation is not known and therapeutically untapped [2]. In vitro, they are fibroblast-like cells with a great ability to adhere to plastic and they display a specific phenotype. The immunological profile of MSCs should be carefully evaluated, particularly regarding their immunogenicity. MSCs can be virtually found in almost all tissues, which makes easy their isolation and expansion. The sources of MSCs must be acknowledged and optimized to ensure their appropriate identity and properties. However, there are still many limitations and unresolved problems regarding stem cell therapy in terms of ethical barriers, immune rejection, tumorigenicity, and cell sources [3]. Depending on their origin, they can exert several functions including support of hematopoiesis, tissue repair, immunomodulation, and the stimulation of resident progenitor cells. Although these cells possess robust therapeutic properties that can be applied in the treatment of different diseases, variables in preclinical and clinical trials lead to inconsistent outcomes [4]. Historically applied for regenerative medicine, the capacity of MSCs to differentiate into multiple lineages was linked to their therapeutic function. As proposed, they may act as trophic and anti-inflammatory signaling cells rather than by differentiating cells. MSCs are not immune cells but tissue precursor cells with immunomodulatory features that show important interplay and interactions with other tissue progenitor cells. It is likely that the beneficial effects of MSCs result from their paracrine pathways affected by either local environment or culture conditions [5]. Indeed, MSCs, by sensing different signals within the local tissue, are environmentally responsive cells that show plasticity in their functions. The influence of the environment allows MSCs to adapt their fate when facing tissue challenges. It is now recognized that they release different mediators including growth factors, cytokines, and genetic material underlying the therapeutic effects of MSCs. These molecules may be secreted or packaged within extracellular vesicles (EVs). EVs are nano-sized membrane-bound vesicles that shuttle important signals between cells to maintain physiological homeostasis. By selectively isolating these MSC-derived vesicles, they can be infused instead of the cells for different clinical purposes [6]. EVs not only have the same effects as MSCs, but they also have the advantages of targeted delivery, low immunogenicity, and high repairability. Pre-clinical studies using animal models of disease have been conducted with MSC-derived EVs. The effectiveness of these strategies is associated with the presence of several parameters that might impact the quantity and quality of the released EVs. The mechanism of action of EVs, which is required for translating preclinical data to clinics, remains to be determined [7]. Whereas many characteristics of MSCs have been reported, other aspects of their biological, immunological, and trophic features during tissue healing need to be more clarified. All these features will contribute in improving the safety and efficiency of MSC-based therapy.
